# Multilayered molecular mechanisms and regulatory networks of aminoglycoside resistance in Enterobacteriaceae

**DOI:** 10.3389/fcimb.2026.1967050

**Published:** 2026-09-23

**Authors:** Yiting Lin, Ling Peng, Shuojie Lv, Wanying Zheng, Qingjing Wang

**Affiliations:** 1 Key Laboratory of Artificial Organs and Computational Medicine of Zhejiang Province, Key Laboratory of Pollution Exposure and Health Intervention of Zhejiang Province, Shulan International Medical College, Zhejiang Shuren University, Hangzhou, China; 2 Hangzhou Cining Hospital, Hangzhou, China

**Keywords:** aminoglycosides, antimicrobial resistance, Enterobacteriaceae, heteroresistance, mobile genetic elements, resistance mechanisms

## Abstract

Enterobacteriaceae, as common clinical pathogens, pose a significant threat to global public health because of the infections they cause. Aminoglycoside antibiotics are critical for treating these infections; however, increasing antimicrobial resistance severely limits their clinical efficacy. This review provides a synthesis of the molecular mechanisms underlying aminoglycoside resistance in Enterobacteriaceae, including the roles of aminoglycoside-modifying enzymes, high-level resistance mediated by 16S rRNA methylation, alterations in bacterial membrane permeability, and heteroresistance. These resistance mechanisms are typically mediated by mobile genetic elements such as plasmids and integrons, facilitating rapid dissemination among bacterial strains. This review aims to provide a theoretical foundation for optimizing clinical antimicrobial strategies, developing novel therapeutic agents, and establishing effective surveillance frameworks.

## 1 Introduction

Enterobacteriaceae are a family of gram-negative bacteria that include clinically significant pathogens such as *Escherichia coli*, *Klebsiella pneumoniae*, and *Enterobacter cloacae*. They are the primary etiological agents responsible for both community-acquired and hospital-acquired infections, contributing substantially to global morbidity and healthcare burden (Holmes et al., 2021). Aminoglycoside antibiotics exert their potent bactericidal effects by irreversibly binding to the bacterial 30S ribosomal subunit (Kotra et al., 2000), thereby inhibiting protein synthesis. They play a crucial role in treating severe infections caused by multidrug-resistant gram-negative organisms (Thy et al., 2023). However, the increasing challenge of antimicrobial resistance has led to a sustained global increase in aminoglycoside resistance among Enterobacteriaceae. According to the WHO GLASS 2025 report, approximately one in six laboratory-confirmed bacterial infections worldwide were resistant to antibiotic treatment in 2023, with resistance rates increasing by over 40% between 2018 and 2023. Regional disparities were substantial: resistance was highest in the South-East Asia and Eastern Mediterranean regions (one in three infections), followed by the African Region (one in five). Among Gram-negative pathogens, more than 40% of E. coli and over 55% of K. pneumoniae globally are now resistant to third-generation cephalosporins, with resistance exceeding 70% in the African Region (World Health Organization, 2025). Annual deaths attributable to antimicrobial-resistant infections exceed one million, with regional aminoglycoside resistance rates varying widely—for example, exceeding 30% (Murray et al., 2022) in some surveillance studies, consequently increasing treatment failure rates and healthcare costs. A comprehensive understanding of the underlying resistance mechanisms is therefore imperative for addressing this public health crisis. This review aims to delineate the multilayered molecular mechanisms of aminoglycoside resistance in Enterobacteriaceae, with a focus on resistance gene transmission dynamics and bacterial stress response-mediated adaptive resistance, while also evaluating current detection methodologies to inform precision therapy and novel drug development (Serio et al., 2018).

## 2 Molecular mechanisms of aminoglycoside resistance in Enterobacteriaceae

### 2.1 Acetyltransferases

Among the diverse acquired resistance mechanisms in Enterobacteriaceae, the production of aminoglycoside-modifying enzymes (AMEs) (Foudraine et al., 2021) represents the most classical and prevalent strategy.

#### 2.1.1 Chemical modification leading to antibiotic inactivation

Aminoglycoside acetyltransferases (AACs) catalyze the transfer of an acetyl group from acetyl coenzyme A to specific amino groups on the aminoglycoside molecule, resulting in antibiotic inactivation. This inactivation occurs through two primary mechanisms: charge neutralization and steric hindrance. Aminoglycosides rely on electrostatic interactions between their positively charged amino groups and the negatively charged phosphate backbone of bacterial ribosomal RNA for high-affinity binding. Acetylation converts these positively charged amino groups to neutral acetamido groups, disrupting this essential interaction. Concurrently, the introduced acetyl group creates steric hindrance that interferes with precise drug docking into the ribosomal A-site, thereby abrogating antimicrobial activity.

#### 2.1.2 Acetylated aminoglycosides exhibit reduced ribosomal binding and elevated resistance levels

AAC-mediated acetylation (Bordeleau et al., 2024) alters the charge distribution and spatial conformation of aminoglycoside molecules, diminishing their binding affinity for the 16S rRNA A-site of the 30S ribosomal subunit. This reduced affinity elevates the minimum inhibitory concentration (MIC), leading to clinical resistance. Structural analyses indicate that acetylation sites are typically proximal to key amino groups involved in ribosome binding, directly compromising target interactions. AAC(6′)-Ib (Foudraine et al., 2021) is widely distributed among gram-negative bacteria and constitutes a significant molecular determinant of amikacin resistance. Furthermore, novel AACs such as ApmA exhibit broad substrate specificity, with a single enzyme conferring resistance to multiple aminoglycosides.

AACs are classified by modification site into several families, among which AAC(3) and AAC(6′) are most prevalent (Vakulenko and Mobashery, 2003) in Enterobacteriaceae. These families and their subclasses differ markedly in substrate specificity, which determines selective rather than pan-class cross-resistance. AAC(3) enzymes (e.g., AAC(3)-II, AAC(3)-IV) modify the 3-amino group of the central 2-deoxystreptamine ring and show high activity against gentamicin, tobramycin, and sisomicin but weak activity against amikacin; accordingly, AAC(3)-producing strains typically exhibit gentamicin–tobramycin resistance while remaining susceptible to amikacin (Haldorsen et al., 2014). The AAC(6′) family requires further subcategorization (Casin et al., 1998): AAC(6′)-I enzymes (including AAC(6′)-Ib) target the 6′-amino group of ring I, efficiently modifying amikacin, tobramycin, and netilmicin but exhibiting lower efficiency against gentamicin (Rather et al., 1992); thus, AAC(6′)-I-producing strains typically show amikacin and tobramycin resistance while gentamicin may retain activity. In contrast, AAC(6′)-II enzymes, despite belonging to the same family, display a markedly different substrate profile—efficiently modifying gentamicin, tobramycin, and netilmicin while showing minimal activity against amikacin and isepamicin (Shaw et al., 1989) —resulting in a gentamicin-resistant but amikacin-susceptible phenotype that resembles that of AAC(3) producers but is achieved through a distinct mechanism. Notably, certain bifunctional enzymes, such as AAC(3)-Ib/AAC(6′)-Ib′, have been shown to expand substrate range through the fusion of two catalytic domains, thereby overcoming the substrate limitations of individual subclasses (Kim et al., 2007). This subclass- and enzyme-level substrate diversity directly shapes clinical resistance profiles and is of practical importance when selecting between amikacin and gentamicin in empirical therapy.

A study of 628 community-acquired uropathogenic Enterobacterales from Casablanca, Morocco, reported aminoglycoside resistance rates of 33.5% for gentamicin, 35% for tobramycin, and 21.3% for amikacin. Among AME genes, AAC(3′)-IIa (27.7%) and AAC(6′)-Ib (25.9%) were the most prevalent, while no 16S rRNA methyltransferase genes were detected, suggesting that AMEs remain the primary resistance mechanism in this community setting (Essalhi et al., 2024).

### 2.2 Adenylyltransferases (nucleotidyltransferases)

Aminoglycoside adenylyltransferases (ANTs) (Qi et al., 2024) inactivate antibiotics by covalently transferring a nucleotide moiety, typically AMP, to specific hydroxyl groups on the aminoglycoside molecule. Genes encoding these enzymes, including aadA (conferring streptomycin and spectinomycin resistance) and *ant(4’)-IIa* (conferring tobramycin and amikacin resistance), are widely disseminated on plasmids and integrons. Adenyylation introduces a bulky AMP group, creating steric hindrance that physically obstructs drug entry into the ribosomal A-site while simultaneously altering the net charge and conformation of the molecule, thereby weakening interactions with the target RNA. For example, ANT(2″)-Ia efficiently modifies gentamicin and tobramycin, resulting in high-level resistance.

### 2.3 Phosphotransferases

Aminoglycoside phosphotransferases (APHs) represent another class of AMEs (Wright and Thompson, 1999) widely distributed among bacteria. These enzymes utilize ATP as a phosphate donor to transfer a phosphoryl group to specific hydroxyl residues on the aminoglycoside molecule. This phosphorylation alters the chemical structure of the antibiotic, precluding its binding to the bacterial ribosome and subsequent inhibition of protein synthesis. APH-encoding genes are frequently located on mobile genetic elements, facilitating their horizontal dissemination (Wright and Thompson, 1999; Lin et al., 2024).

### 2.4 16S rRNA methyltransferases

Plasmid-mediated 16S rRNA methyltransferases constitute one of the most clinically significant mechanisms conferring high-level, pan-aminoglycoside resistance in Enterobacteriaceae. These enzymes catalyze the methylation of a specific residue within the 16S rRNA A-site, predominantly G1405. This methylation create ssteric hindrance that confers very high-level resistance to most clinically relevant 4,6-disubstituted deoxystreptamine aminoglycosides, including gentamicin, tobramycin, and amikacin, with MICs typically ≥256 μg/mL for G1405-methylating enzymes such as ArmA, RmtB, and RmtC, as previously established as a hallmark of this resistance mechanism (Al Sheikh et al., 2014; Taylor et al., 2018; Yang and Hu, 2022).

#### 2.4.1 *armA*, *rmtB*, and *rmtC*: key drivers of high-level resistance

*armA*, *rmtB (*Hameed et al., 2026*)*, and *rmtC (*Foudraine et al., 2021*)* represent the most prevalent and clinically significant 16S rRNA methyltransferase genes in Enterobacteriaceae. The encoded enzymes specifically catalyze N7 methylation of guanine at position 1405 (G1405) within the 16S rRNA, conferring extremely high-level resistance (MICs typically ≥256 μg/mL) to the vast majority of clinically relevant aminoglycosides. The epidemiological and clinical characteristics of these enzymes, including their geographic distribution, common co-resistance enzymes, major plasmid types, co-occurring carbapenemases, and plasmid resistance gene linkage patterns, are summarized in **Table 1**.

**Table 1:** Three major 16S rRNA methyltransferases (ArmA, RmtB, and RmtC).

Transferase characteristics	ArmA	RmtB	RmtC
Geographic distribution	Worldwide prevalence	Predominant in Greece and other regions	Sporadic, possibly underestimated
Common coresistance enzymes	Associated with NDM	Associated with KPC/VIM	Associated with NDM-1 and CTX-M-type ESBLs
Major plasmid types	Conjugative plasmid	IncC-type plasmid	Plasmid-mediated
Major co-occurring carbapenemases	NDM-type	KPC/VIM-type	NDM-1/VIM-1-type
Complexity of plasmid resistance gene linkage	Forms multigene complex	Carries multiple ESBLs/carbapenemases	Colocated with blaNDM-1 and other carbapenems and ESBL genes.
References	(Galimand et al., 2005; Fernandez et al., 2022)	(Galani et al., 2012; Roch et al., 2021)	(Guven Gokmen et al., 2016; Zeng et al., 2023)

#### 2.4.2 *rmtF* mediates high-level aminoglycoside resistance

*rmtF* (Foudraine et al., 2021*)* encodes a 16S rRNA methyltransferase that functions analogously to ArmA, RmtB, and RmtC, conferring resistance through G1405 methylation. rmtF is frequently colocalized with NDM carbapenemase genes on diverse plasmid backbones, demonstrating significant potential for dissemination among multidrug-resistant strains (Hidalgo et al., 2013; Sabtcheva et al., 2024). Isolates harboring *rmtF* may also exhibit resistance to last-line agents such as tigecycline and colistin, further complicating therapeutic management (Hidalgo et al., 2013).

#### 2.4.3 NpmA and A1408 methylation: expanding the resistance spectrum

In addition to the G1405 methyltransferases, methylation of adenine at position 1408 (A1408) within the 16S rRNA A-site represents a distinct mechanism that confers resistance to a different subset of aminoglycosides. The NpmA enzyme catalyzes N1 methylation of A1408. Unlike G1405 modification, which primarily affects 4,6-disubstituted deoxystreptamine (DOS) aminoglycosides, A1408 methylation blocks the binding of 4,5-disubstituted DOS agents (e.g., neomycin, paromomycin) and the 4-monosubstituted aminoglycoside apramycin, while also affecting certain 4,6-disubstituted agents (Wachino et al., 2007; Dey et al., 2025). This modification thereby extends the pan-aminoglycoside resistance phenotype to include drugs not affected by G1405 methylation alone. NpmA was first identified in a clinical E. coli isolate from Japan and is plasmid-encoded, indicating its potential for horizontal dissemination (Wachino et al., 2007). Although npmA is less prevalent than armA or rmtB, its presence among Enterobacteriaceae highlights the diversity of 16S rRNA methylation strategies and the ongoing evolution of high-level aminoglycoside resistance.

### 2.5 Synergistic interactions between intrinsic and acquired resistance mechanisms and their transmission basis

The genes encoding the aforementioned aminoglycoside-modifying enzymes and 16S rRNA methyltransferases are typically situated on mobile genetic elements—plasmids (Lopez et al., 2021), transposons, and integrons—enabling horizontal transfer and coselection of multidrug resistance phenotypes (discussed in detail in Section 3).

Beyond genetic mobility, intrinsic stress response systems (CpxRA (Raivio, 2005), Rcs, and the ppGpp-mediated stringent response) are activated by subinhibitory aminoglycoside concentrations. This activation reduces membrane permeability (e.g., via OmpF downregulation) and promotes persister formation, thereby lowering the fitness cost of plasmid carriage and favoring the selection of resistant strains under antibiotic pressure. Notably, plasmid carriage imposes a metabolic burden on hosts through replication and overexpression of resistance genes; compensatory mutations in chromosomal or plasmid genes can mitigate this cost and stabilize plasmids even in the absence of antibiotic selection. These stress-induced adaptations create a permissive environment for the establishment and expression of acquired resistance genes (elaborated in Section 4). Understanding this synergy offers opportunities for developing adjuvants that target bacterial stress pathways.

## 3 Integrons and mobile genetic elements

### 3.1 Integrons

Integrons (Blanco et al., 2024) are genetic platforms capable of capturing, integrating, and expressing exogenous gene cassettes via site-specific recombination, representing a major driver of rapid evolution of multidrug resistance in gram-negative bacteria.

#### 3.1.1 Class 1 integrons

Class 1 integrons (Qi et al., 2023) are closely associated with multidrug resistance in Enterobacteriaceae and are commonly detected in clinical isolates such as *Escherichia coli* and *Klebsiella pneumoniae (*Wang et al., 2022; Wang et al., 2024*)*, with a detection rate reaching 64.38% (Wang et al., 2024) in carbapenem-resistant strains. In a South Indian study of 60 multidrug-resistant Gram-negative clinical isolates, the overall prevalence of the intI1 gene was 60%, with significantly higher detection rates in MDR isolates (80%) compared to non-MDR isolates (43.34%) (Perumal et al., 2024). These integrons carry gene cassettes that confer resistance to aminoglycosides and trimethoprim and are frequently linked to extended-spectrum β-lactamase production. Structurally, class 1 integrons consist of a 5′ conserved segment (5′-CS), a variable region, and a 3′ conserved segment (3′-CS). The 5′-CS contains the *intI1* gene encoding integrase, the attI1 recombination site (Gravel et al., 1998; Demarre et al., 2007), and the Pc and P2 promoters. Transcription of intI1 is repressed by the SOS response regulator LexA, which binds to a LexA box overlapping the intI1 promoter (Guérin et al., 2011). Upon DNA damage caused by antibiotic exposure (e.g., fluoroquinolones, β-lactams) or environmental stress, the SOS response is activated, leading to autocleavage of the LexA repressor and a subsequent increase in intI1 transcription. This regulatory link ensures that integrase expression and cassette recombination are induced primarily under stress conditions, while remaining repressed during steady-state growth to minimize the fitness cost associated with constitutive integrase activity. The variable region is responsible for gene cassette capture and expression, exhibiting a proximal advantage effect whereby cassettes closer to the Pc promoter are expressed at higher levels. 3′-CS carries the *sul1* gene for sulfonamide resistance and *qacEdelta1 (*Chen et al., 2023; Han et al., 2025*)* for disinfectant resistance. Gene cassettes are flanked by attC sites (Mukhortava et al., 2019) that form hairpin structures recognized by the integrase; *IntI1* then mediates site-specific recombination between *attI1* and attC, enabling cassette capture and excision as the core function of integrons. These integrons are often located on conjugative plasmids, and their mobility is further enhanced by insertion sequences such as IS26 (Harmer and Hall, 2024), which facilitates horizontal transfer of resistance genes. Consequently, the strong association between class 1 integrons and multidrug resistance makes them important targets for novel antimicrobial strategies such as CRISPR-Cas9 (**Figure 1**).

**Figure 1:**
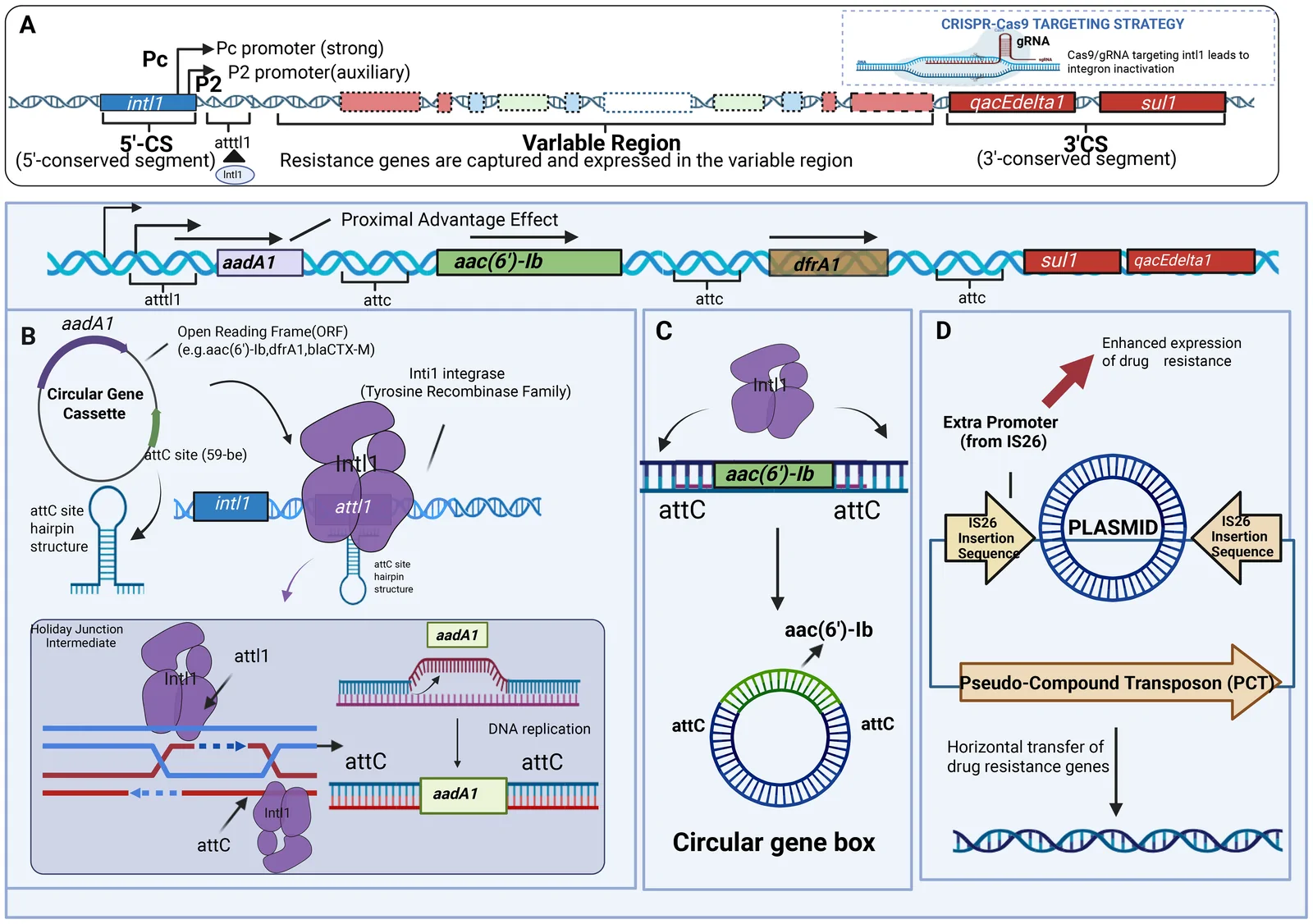
Structure and function of class 1 integrons and associated mobile genetic elements. This figure depicts the overall process of class 1 integron structure, gene cassette capture, excision and circularization, as well as IS26-mediated enhancement and horizontal transfer of resistance genes. **(A)** Class 1 integrons are composed of a 5′-conserved segment (5′-CS) harboring *intI1*, *attI1* and promoters Pc and P2, a variable region, and a 3′-conserved segment (3′-CS) containing qacEdelta1 and *sul1*. A CRISPR-Cas9 strategy targeting *intI1* is indicated. **(B)** IntI1 catalyzes site-specific recombination between the attC site (59-base element, 59-be) of the circular *aadA1* gene cassette and *attI1*, forming a Holliday junction. The cassette is stably integrated into the variable region following DNA replication. **(C)** The integrated *aac(6′)-Ib* gene cassette is excised and circularized via intramolecular recombination between flanking attC sites to form a free circular molecule. **(D)** IS26 elevates resistance gene expression using its own promoter and forms pseudo-compound transposons (PCT) to drive horizontal transfer of resistance genes in bacterial genomes.

#### 3.1.2 aac(6′)-Ib

The *aac(6′)-Ib* gene (Raherison et al., 2017) is captured as a resistance gene cassette by class 1 integrons and integrated into the variable region, thereby contributing to multidrug resistance. This gene encodes an aminoglycoside 6′-N-acetyltransferase that transfers an acetyl group from acetyl-CoA to the 6′ amino position of the antibiotic, thus inactivating the drug. The cassette is flanked by attC recombination sites that form hairpin structures recognized by the IntI1 integrase, allowing stable insertion into the variable region via site-specific recombination. Moreover, the repeated attC sites can undergo intramolecular recombination, mediating excision and circularization of the cassette to form a free mobile cassette and thereby confer horizontal transfer potential. Given these features, *aac(6′)-Ib* is widely prevalent in gram-negative pathogens and serves as a core determinant of resistance to clinically critical aminoglycosides such as amikacin. Notably, its variant *aac(6′)-Ib-cr* additionally has the ability to modify fluoroquinolones, further broadening the resistance profile. Through a mobile genetic system composed of integrons and IS26, both integrated and free forms of *aac(6′)-Ib* can spread rapidly among strains, driving the dissemination of multidrug resistance in clinical Enterobacteriaceae (**Figure 1**).

#### 3.1.3 aadA

The *aadA* gene encodes an aminoglycoside adenyltransferase that transfers an AMP moiety to a specific hydroxyl group of the aminoglycoside molecule, thereby modifying and inactivating the antibiotic and conferring resistance. This gene exists as a resistance cassette flanked by attC hairpin structures recognized by the *IntI1* integrase, which enables its stable integration into the variable region of class 1 integrons via site-specific recombination. *aadA* primarily mediates resistance to streptomycin and spectinomycin and is frequently located on mobile genetic elements. Leveraging the capture and excision properties of integrons, this cassette undergoes horizontal transmission among strains, representing a major driver of aminoglycoside resistance in clinical Enterobacteriaceae (**Figure 1**).

#### 3.1.4 Insertion sequences

Insertion sequences (Couchoud et al., 2020) are frequently colocalized with antibiotic resistance genes in Enterobacteriaceae and promote aminoglycoside resistance through two major mechanisms: facilitating horizontal gene transfer and enhancing resistance gene expression. On the one hand, IS elements can assemble into pseudocompound transposons or transposition units, thereby mediating the horizontal transfer of resistance genes between chromosomes and plasmids and accelerating their dissemination within bacterial populations. Notably, resistance genes encoding aminoglycoside-modifying enzymes frequently colocalize with IS elements on mobile genetic elements, further increasing transmission efficiency. On the other hand, IS elements carry their own promoters, which can upregulate the transcription of adjacent resistance genes and significantly potentiate the resistance phenotype. Overall, IS elements play a central role in driving the spread and expression of aminoglycoside resistance in Enterobacteriaceae (Wang et al., 2024) (**Figure 1**).

### 3.2 Transposons

Transposons are genetic elements capable of moving between different DNA molecules within a bacterial genome, serving as critical vectors for resistance gene rearrangement and horizontal dissemination.

#### 3.2.1 Tn3

Tn3 (Shkumatov et al., 2022) family transposons represent important DNA transposition elements in Enterobacteriaceae. They utilize a replicative transposition mechanism dependent on the transposase TnpA and resolvase TnpR to replicate and insert themselves along with associated resistance genes, enhancing gene mobility between plasmids and chromosomes. These transposons frequently carry β-lactamase genes (Coertze and Bezuidenhout, 2019) and may also harbor aminoglycoside resistance genes, promoting their horizontal transfer through transpositional activity. Additionally, they can introduce promoters that activate the expression of otherwise silent resistance genes. Tn3 family transposons commonly interact synergistically with integrons and insertion sequences to assemble complex resistance islands, driving the rapid evolution and dissemination of multidrug resistance in Enterobacteriaceae (Brovedan et al., 2021).

#### 3.2.2 Tn7

Tn7 family transposons (Hsieh and Peters, 2024) are characterized by highly specific target site selection and a complex multicomponent transposition system comprising five core transposition genes (tnsA, tnsB, tnsC, tnsD, tnsE) and a cargo region typically carrying resistance genes. Tn7 utilizes two distinct target-selection pathways: the TnsD pathway directs integration into the conserved chromosomal attTn7 site downstream of gene (Traykovska et al., 2021), ensuring stable vertical inheritance; the TnsE pathway preferentially targets conjugative plasmids and actively replicating DNA, enabling horizontal dissemination. This dual-pathway architecture balances vertical stability with horizontal mobility. For aminoglycoside resistance, Tn7 and its derivatives frequently carry class 1 integrons containing aadA gene cassettes within their cargo regions, enabling stable expression of aadA via TnsD while also facilitating its spread via TnsE. Tn7 thus constitutes a versatile mechanism for both long-term persistence and intercellular dissemination of aminoglycoside resistance genes, particularly those conferring streptomycin and spectinomycin resistance, within Enterobacteriaceae populations.The functional characteristics of the five core Tn7 proteins, including their DNA cleavage activities, unique enzymatic features, target recognition sites, and regulatory mechanisms, are detailed in **Table 2**.

**Table 2:** Functional characteristics of core proteins of the Tn7 family of transposons.

Feature	TnsA	TnsB	TnsC	TnsD	TnsE
Functional characteristics	Cleavage of 5’ terminal DNA	Cleavage of 3’ terminal DNA and strand transfer	Regulates transposition, recruits TnsAB	Recognizes the attTn7 safe site	Recognizes replication fork, facilitates horizontal transfer
Unique feature	Restriction endonuclease-like	DDE catalytic motif	ATPase, mediates target immunity	Sequence-specific binding	Structure-specific DNA binding
Target site/recognition object	Outside of 5’ transposon end	Left and right terminal sequences of transposon	Target DNA and TnsD/E	Downstream of glmS gene (attTn7)	DNA replication fork structure
Regulatory mechanism	Forms heterodimer with TnsB	Cooperates with TnsA for transposition	ATP hydrolysis drives conformational change	Interacts with TnsC to guide insertion	Preferentially inserts into conjugative plasmids
References	(Sarnovsky et al., 1996; Peters, 2014)	(Sarnovsky et al., 1996; Peters, 2014)	(Stellwagen and Craig, 1997; Peters, 2014; Leyva Camacho et al., 2025)	(Parks and Peters, 2007; Peters, 2014; Leyva Camacho et al., 2025)	(Parks and Peters, 2007; Peters, 2014; Krishnan et al., 2025)

## 4 Heteroresistance and stress responses

Heteroresistance (Andersson et al., 2019) refers to the phenomenon wherein a bacterial population derived from a single clone presents a minor subpopulation that is resistant to an antibiotic, whereas the majority remain susceptible, a phenomenon distinct from conventional resistance.

### 4.1 The CpxRA two-component system

Aminoglycosides enter the periplasm via OmpF porins, impair protein synthesis, and cause misfolded protein accumulation, which activates the CpxRA system (Raivio, 2005). Activated CpxR then represses ompF transcription (Batchelor et al., 2005) (with H-NS (Suzuki et al., 1996)) and upregulates chaperones/proteases (Spy/DegP*).* CpxRA activation can also reduce the proton motive force (PMF), diminishing the electrochemical gradient (Δψ) that drives aminoglycoside uptake across the inner membrane (Choi et al., 2024; Choe et al., 2025). CRISPRi screening and transcriptomic analyses have shown that CpxR directly regulates genes involved in respiratory complexes, including NADH-quinone oxidoreductase and succinate dehydrogenase, leading to membrane depolarization under gentamicin exposure (Tsviklist et al., 2021; Choe et al., 2025).These layered responses limit drug influx, alleviate proteotoxicity, and foster a subpopulation capable of surviving high aminoglycoside concentrations, thus contributing to heteroresistance (Choi et al., 2024).This is supported by evidence that cpxR inactivation increases gentamicin susceptibility in E. coli and Salmonella (Huang et al., 2016), whereas constitutive Cpx activation raises MICs (Choi et al., 2024) (**Figure 2**).

**Figure 2:**
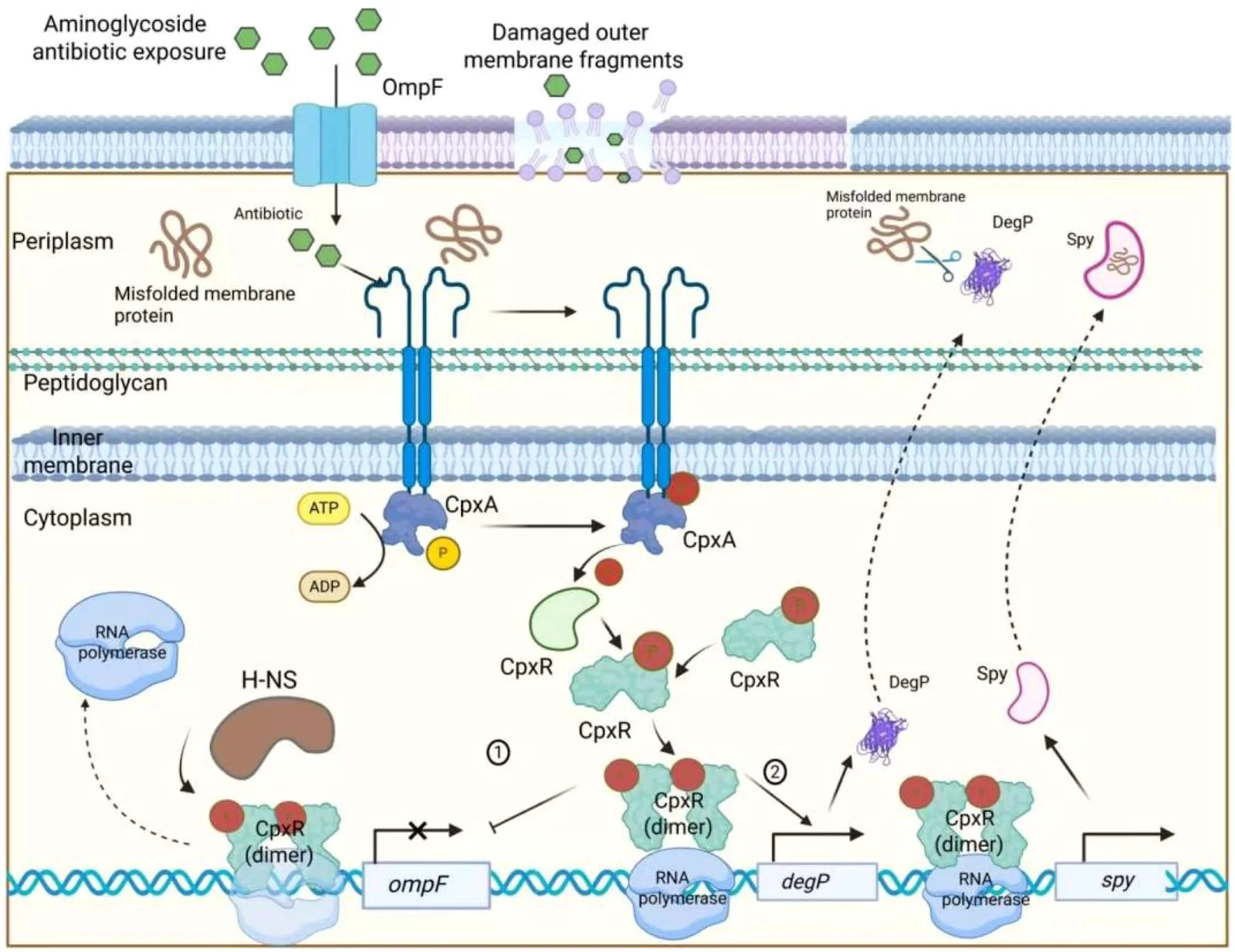
Mechanism of the Cpx two-component system mediating bacterial heteroresistance to aminoglycosides.The CpxRA two-component system senses envelope integrity and periplasmic protein folding stress in bacteria. Aminoglycosides disrupt the outer membrane and enter the periplasm via outer membrane protein F (OmpF), disturbing protein synthesis, accumulating misfolded proteins and aggravating envelope damage. The transmembrane histidine kinase CpxA autophosphorylates upon sensing stress signals and transfers phosphate to the cognate response regulator CpxR. Phosphorylated CpxR forms homodimers and acts as a transcription factor with dual functions. CpxR directly represses ompF transcription, while H-NS independently downregulates OmpF expression via the micF antisense RNA pathway, decreasing porin production and reducing antibiotic influx.Meanwhile, it upregulates *degP* and *spy* The periplasmic protease DegP degrades toxic misfolded proteins, and the molecular chaperone Spy promotes protein refolding. Together they relieve cellular toxicity and maintain envelope homeostasis. This adaptive mechanism allows bacterial subpopulations to survive high antibiotic concentrations and develop heteroresistance.

### 4.2 The Rcs phosphorelay system

The Rcs (Regulator of Capsule Synthesis) system is a multistep phosphorelay signal transduction pathway that is primarily responsible for monitoring and maintaining the integrity of the outer cell envelope layers (Wall et al., 2020).The Rcs cascade proceeds through RcsC autophosphorylation, phosphotransfer to the intermediate RcsD, and finally to the response regulator RcsB (Takeda et al., 2001).Upon activation, phosphorylated RcsB promotes the expression of genes involved in capsular polysaccharide (CPS) synthesis, increasing capsule thickness and density. This enhanced capsule layer can physically shield and electrostatically repel positively charged aminoglycoside molecules, reducing drug access to the cell surface. Importantly, the protective efficacy of Rcs-mediated capsule upregulation may vary across Enterobacteriaceae species. Klebsiella pneumoniae naturally produces a thick hypermucoviscous capsule, and RcsB has been shown to activate Klebsiella K2 capsule synthesis in E. coli (Wacharotayankun et al., 1992), whereas E. coli maintains a thinner basal capsule layer (Amako et al., 1988). This baseline difference suggests that Rcs activation may confer a greater relative increase in aminoglycoside protection in K. pneumoniae than in E. coli (Walker et al., 2019). The Rcs system thus provides a more specialized defense strategy that may contribute to heteroresistance by modifying cell surface physicochemical properties in response to specific environmental cues.The composition, activation signals, signal transduction pathway, effector responses, resistance mechanisms, and specificity of the Rcs phosphorelay system are summarized in **Table 3**.

**Table 3:** Composition and function of the Rcs phosphorelay system.

Rcs characteristic dimension	Core components/signals	Significance	Reference
Composition	RcsF, RcsC, RcsD, RcsB; multistep phosphorelay	Enables signal integration and stepwise transduction	(Zhang and Shi, 2005; Li et al., 2024)
Activation	Responds to periplasmic/cell width changes and antibiotic stress	Specifically monitors envelope integrity	(Zietek et al., 2025)
Signal transduction	Periplasmic thinning brings RcsF to inner membrane, activating cascade	Converts physical damage into chemical signal	(Zietek et al., 2025)
Effector response	RcsB activates capsule synthesis and stress genes	Remodels cell surface to adapt to environmental stress	(Li et al., 2024)
Resistance mechanism	Capsule thickening may reduce drug penetration; negative charge may contribute to aminoglycoside exclusion; may regulate FtsZ	May contribute to heteroresistance	(Campos et al., 2004; Wall et al., 2018)
Specificity	Senses cell width/periplasmic thickness; distinct from CpxRA	Specific defense, complementary to CpxRA	(Zietek et al., 2025)

### 4.3 The stringent response (ppGpp-mediated)

The stringent response represents a conserved bacterial stress adaptation that, during aminoglycoside exposure, reprograms transcription, translation, and membrane permeability to promote survival.

#### 4.3.1 Transcriptional reprogramming

ppGpp orchestrates a bifunctional transcriptional program: it represses rRNA and tRNA transcription to arrest growth (Paul et al., 2004), while can induce expression of stress regulators including marA and SoxS.MarA and SoxS then upregulate the acrAB-tolC (Duval and Lister, 2013) multidrug efflux pump genes, driving active extrusion of aminoglycosides.This ppGpp–MarA/SoxS–AcrAB-TolC axis represents a direct regulatory link between the stringent response and aminoglycoside resistance.

#### 4.3.2 Ribosome regulation and translational inhibition

(p)ppGpp suppresses global translation at multiple levels (Bennison et al., 2019).This suppression directly counteracts the bactericidal mechanism of aminoglycosides, which depends on binding to actively translating ribosomes to induce miscoding and lethal misfolded protein production.By reducing overall translation activity, (p)ppGpp diminishes the frequency of drug-induced miscoding events and toxic protein accumulation, thereby conferring tolerance (**Figure 3**).

**Figure 3:**
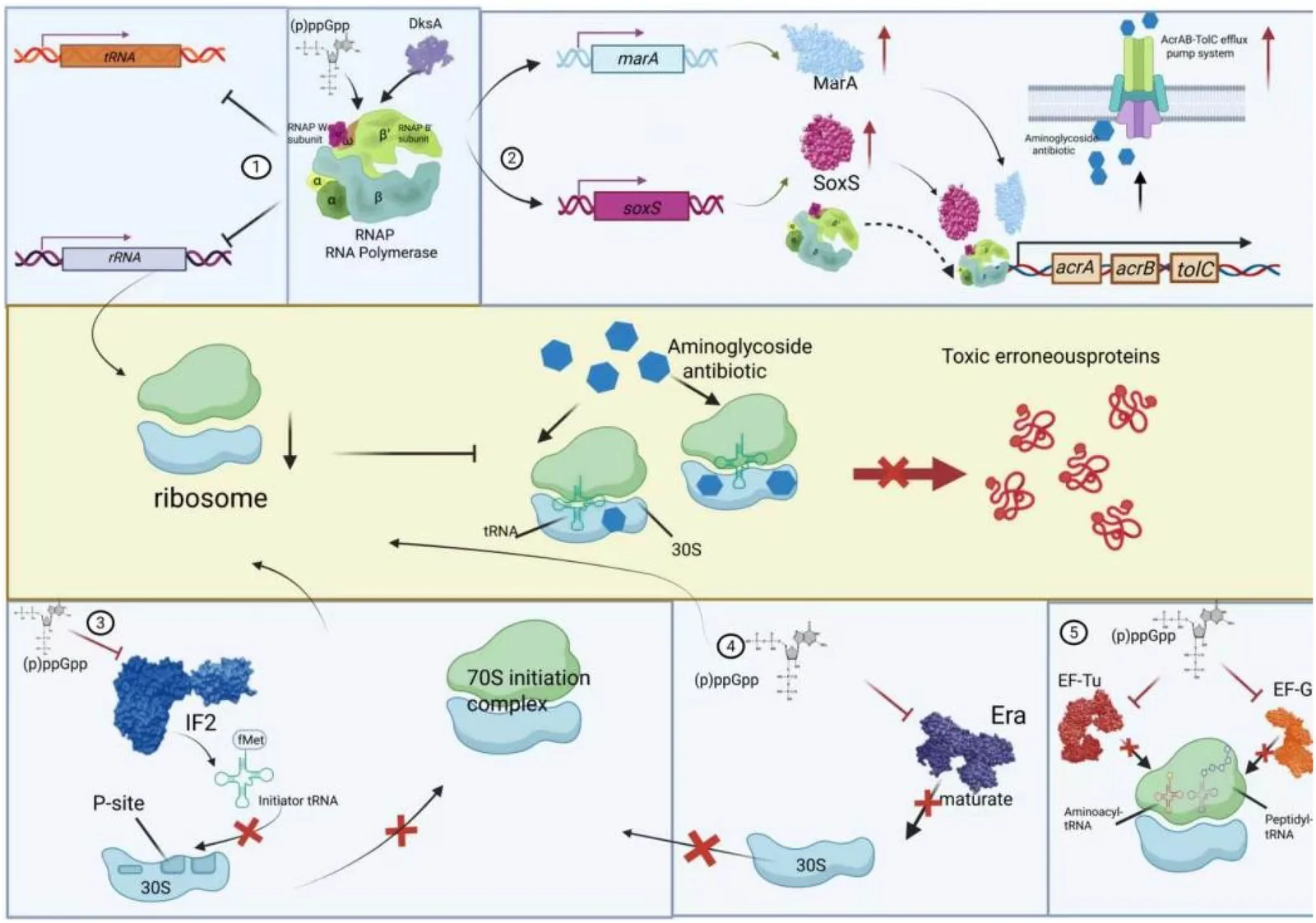
Mechanisms of bacterial heteroresistance to aminoglycosides.Bacteria activate the stringent response under aminoglycoside antibiotic stress, with the core regulatory molecule (p)ppGpp (guanosine tetraphosphate and pentaphosphate) mediating heteroresistance through coordinated transcriptional and translational regulation. (p)ppGpp, in synergy with DksA (DnaK suppressor A), remodels RNA polymerase (RNAP) to exert dual effects: on one hand, it represses the transcription of rRNA (ribosomal RNA) and tRNA (transfer RNA), reducing the abundance of translation machinery; on the other hand, it activates the expression of *marA* (multiple antibiotic resistance A) and *soxS* (superoxide response S), thereby upregulating the AcrAB-TolC efflux pump system to enhance drug extrusion. Meanwhile, (p)ppGpp targets key steps in translation: inhibiting IF2 (initiation factor 2) to block initiation complex assembly, interfering with Era (E. coli Ras-like protein A) to hinder 30S ribosomal subunit maturation, and suppressing EF-Tu (elongation factor Tu)/EF-G (elongation factor G) to disrupt peptide chain elongation. These multi-layered regulations collectively reduce translational activity, diminish the production of toxic mistranslated proteins induced by aminoglycosides, and promote bacterial survival under antibiotic pressure.

#### 4.3.3 Membrane function and drug permeability modulation

ppGpp upregulates MicF, which suppresses OmpF and OmpC.These porins are the primary entry routes for hydrophilic aminoglycosides, so their downregulation limits drug influx (Bafna et al., 2020). Concomitantly, ppGpp alters membrane phospholipid composition (e.g., increasing cardiolipin), reducing membrane fluidity (Traxler et al., 2008).Together with efflux pump activation, these changes create a dual-barrier defense—reduced influx and enhanced efflux—that lowers intracellular aminoglycoside accumulation.

#### 4.3.4 ppGpp-mediated persister cell formation and tolerance mechanisms

The stringent response drives a subpopulation of bacteria into a dormant persister state, a process in which (p)ppGpp plays an essential role in mediating tolerance to antibiotics and the formation of persister cells (Bakkeren et al., 2019). Persister cells exhibit two hallmark features that directly counter aminoglycoside action: proton motive force (PMF) collapse, which precludes PMF-dependent drug uptake, and translational arrest, which eliminates the active ribosomes required for aminoglycoside-induced miscoding.

Downstream of ppGpp, three interconnected mechanisms enforce this dormancy. First, toxin-antitoxin (TA) modules such as HipBA and TisB/IstR are activated. Type II TA systems like HipBA are well-established persistence determinants, while the type I system TisB/IstR is activated under SOS-inducing conditions (Dörr et al., 2010; Nonin-Lecomte et al., 2021).Upon expression, TisB inserts into the inner membrane, causing the collapse of PMF and a drop in ATP levels, which induces a dormant state that protects cells from aminoglycoside killing.HipA-induced persistence depends not only on (p)ppGpp but also on TA-encoded mRNases. Second, (p)ppGpp-mediated GTP depletion limits energy supply for translation and other essential processes (Hauryliuk et al., 2015). Third, the SOS response induces additional TA modules such as TisB/IstR, reinforcing dormancy (Dörr et al., 2010).

These TA-mediated and GTP-depletion effects synergize with ppGpp to maintain deep dormancy.Importantly, the persister subpopulation may serve as a reservoir for horizontal acquisition of resistance plasmids (Bakkeren et al., 2019).Recent studies have shown that tissue-associated persisters of Salmonella enterica represent long-lived reservoirs of plasmid donors or recipients, and the re-seeding of these persister bacteria into the gut lumen enables plasmid transfer between various strains of Enterobacteriaceae (Bakkeren et al., 2021).This suggests that persisters can facilitate the transition from phenotypic tolerance to stable genetic resistance, although this process is likely context-dependent rather than inevitable (Windels et al., 2019).

## 5 Resistance transmission and detection

### 5.1 Horizontal gene transfer mechanisms

The rapid dissemination of resistance genes within bacterial populations relies primarily on three horizontal gene transfer mechanisms: transformation, conjugation, and transduction (Vos et al., 2024).Transformation involves bacterial uptake and chromosomal integration of extracellular DNA fragments; although it occurs at a relatively low frequency in Enterobacteriaceae (Winter et al., 2021), it retains significance under specific conditions. Conjugation constitutes the most efficient and influential pathway within Enterobacteriaceae, requiring direct cell–to–cell contact mediated by mobile genetic elements, predominantly conjugative plasmids. Sex pili mediate donor–recipient cell pairing, following which plasmid DNA is transferred as a single strand through a type IV secretion system, subsequently completing complementary strand synthesis and recircularization within the recipient. This process equips the recipient with the full plasmid-encoded resistance gene complement, converting it into a potential new donor and establishing exponential resistance propagation. Transduction employs bacteriophages as vectors; phages may erroneously package resistance gene fragments during infection of donor bacteria, subsequently injecting these genes upon infecting new susceptible hosts. Certain phage–plasmid elements that exhibit dual characteristics can carry multiple clinically significant resistance genes, including aminoglycoside resistance determinants, and can convert susceptible bacteria through infection. Transduction operates independently of direct cellular contact, enabling resistance gene dissemination across temporal and spatial barriers (Cadamuro et al., 2025).

### 5.2 Overview and comparison of major detection methods

#### 5.2.1 Phenotypic methods

Traditional antimicrobial susceptibility testing, exemplified by broth microdilution and disk diffusion methods, remains the gold standard in clinical microbiology laboratories. These approaches determine minimum inhibitory concentrations (MICs) or inhibition zone diameters by assessing bacterial growth in the presence of defined antibiotic concentrations, providing direct readouts of susceptibility or resistance. While offering the advantages of direct clinical relevance and standardization, phenotypic methods are time-consuming (typically requiring 16–24 hours), fail to elucidate specific molecular resistance mechanisms, and exhibit limited sensitivity for detecting heteroresistant and persister phenotypes (Band and Weiss, 2019; Salam et al., 2023).

#### 5.2.2 Molecular methods

Polymerase chain reaction (PCR)-based techniques and their derivatives enable direct detection of specific resistance genes in clinical samples or colonies. For aminoglycoside resistance, targeted assays employ specific primers and probes to detect common modifying enzyme-encoding genes (e.g., *aac(6′)-Ib*, *aph(3′)-VI*, *ant(2″)-Ia*) and 16S rRNA methyltransferase genes (e.g., *armA*, *rmtB)*. These methods offer the advantages of rapid turnaround (hours), high sensitivity, and specificity; for example, novel multiplex fluorescence PCR assays can simultaneously detect seven aminoglycoside resistance genes with detection limits as low as 47–189 copies per reaction. Limitations include restrictions to known target sequences, potentially missing novel variants, and the inability to confirm whether gene presence correlates with phenotypic expression.

#### 5.2.3 Mass spectrometry

Matrix-assisted (Calderaro and Chezzi, 2024) laser desorption/ionization time-of-flight mass spectrometry (MALDI-TOF MS) is widely established for rapid microbial identification. Resistance detection can indirectly infer enzymatic activity (e.g., β-lactamases) by analyzing characteristic hydrolysis products following antibiotic–bacteria coincubation. Recent integration with machine learning algorithms represents an emerging direction for predicting antibiotic resistance (Calderaro and Chezzi, 2024). Systematic reviews indicate that algorithms such as random forests, support vector machines, and logistic regression achieve >80% accuracy, sensitivity, and specificity in predicting aminoglycoside resistance in *E. coli* using MALDI-TOF MS data. However, direct detection of aminoglycoside-modifying enzyme activity remains technically challenging, and clinical applications in this area are currently less established than those for β-lactamase detection.Despite these promising developments, MALDI-TOF MS has important technical limitations that constrain its application in aminoglycoside resistance detection. First, it cannot reliably detect single nucleotide polymorphisms (SNPs) that confer resistance, such as mutations in rpsL, rpsJ, or 16S rRNA genes, as these target sequence alterations do not produce mass shifts detectable by this platform (Calderaro and Chezzi, 2024). Second, it does not readily distinguish between resistance conferred by low-level enzymatic modification (e.g., AME-mediated acetylation or phosphorylation) versus high-level target methylation (e.g., 16S rRNA methyltransferases such as ArmA and RmtB), as both may produce subtle or overlapping spectral changes that are difficult to resolve (Calderaro and Chezzi, 2024). Third, strain-to-strain variability in protein expression profiles and matrix effects can obscure resistance-associated peaks, limiting reproducibility across different laboratories (Paskova et al., 2020). Consequently, MALDI-TOF MS serves as a complement to, rather than a replacement for (Yang et al., 2023), genotypic methods such as PCR and whole-genome sequencing, which remain essential for precise resistance gene identification, SNP detection, and surveillance of emerging resistance determinants.

## 6 Challenges and future perspectives

### 6.1 Current challenges

Aminoglycoside resistance in Enterobacteriaceae presents three major challenges. First, resistance mechanisms exhibit complex synergy; clinical isolates frequently carry multiple modifying enzyme-encoding genes, 16S rRNA methyltransferase genes (detected in up to 63.3% (Li et al., 2024)of carbapenem-resistant Enterobacteriaceae in some surveillance studies), and efflux pump systems, which are intertwined with stress response-mediated adaptive tolerance. In a UK and Ireland surveillance study of 806 pan-aminoglycoside-resistant Enterobacteriaceae, 94.5% carried 16S RMTase genes, with armA being the most common (44.6%), followed by rmtC (19.2%), rmtF (18.0%), and rmtB (11.4%); 93.4% of these also carried acquired carbapenemase genes, predominantly blaNDM (83.1%). A Bulgarian cancer hospital genomic surveillance study (2006–2023) found that 150 (1.4%) of 10,731 Enterobacterales isolates were 16S-RMTase positive, with armA predominating (92.7%), and 97% co-harbored ESBL genes, primarily blaCTX-M-3 (67%) (Sabtcheva et al., 2024). The interactive dynamics among these mechanisms remain incompletely understood, complicating targeted intervention development. Second, existing detection methods have significant limitations: phenotypic testing is time-consuming and inadequately captures heteroresistance; molecular diagnostics are restricted to known resistance genes; and *in vitro* susceptibility results may not accurately predict *in vivo* efficacy within complex infection microenvironments. Third, resistance dissemination poses ecological and epidemiological challenges, with resistance gene reservoirs continuously circulating among human, animal, and environmental microbiomes. Widespread antimicrobial and heavy metal use in agriculture and animal husbandry creates potent coselective pressures within the environmental microbiota (Murray et al., 2024), rendering hospital-focused control measures insufficient to contain the spread of global resistance (Holmes et al., 2016).

### 6.2 Future directions

Addressing these challenges requires coordinated multidimensional efforts. At the fundamental research level, priorities include elucidating the global epidemiology of emerging resistance genes, delineating regulatory networks governing heteroresistance and persister formation, and exploring synergistic interactions among different resistance mechanisms within single strains to identify common vulnerabilities. In diagnostic development, there is an urgent need for rapid, sensitive point-of-care technologies integrating phenotypic and genotypic information to enable early resistance detection and precision antimicrobial guidance, alongside advancing the clinical translation of whole-genome sequencing. Therapeutically, strategies should encompass developing specific inhibitors or adjuvants targeting key resistance mechanisms, exploring metabolic intervention approaches to restore antibiotic efficacy, and devising anti-transmission strategies such as plasmid conjugation inhibitors to curb resistance gene spread at its evolutionary origin. Ultimately, effective containment of this global public health challenge necessitates coordinated action within the “One Health” framework, which integrates the human, animal, and environmental health sectors.

## 7 Conclusion

The resistance of Enterobacteriaceae to aminoglycosides is driven by multiple molecular mechanisms. Enzymatic inactivation by aminoglycoside-modifying enzymes is the most prevalent strategy, whereas plasmid-borne 16S rRNA methyltransferases confer high-level pan-resistance through target site methylation, posing the most serious clinical threat. These resistance genes are located primarily on integrons, transposons, and insertion sequences, facilitating rapid spread via horizontal gene transfer. Moreover, envelope stress response systems and the ppGpp-mediated stringent response remodel membrane permeability, the capsule structure, and metabolic activity, inducing heteroresistance and persister formation, which creates favorable conditions for resistance gene dissemination. The ongoing transmission of resistance genes across human, animal, and environmental reservoirs urgently demands an integrated surveillance system. Future research should elucidate the regulatory network linking adaptive and acquired resistance, develop rapid and accurate diagnostic tools, and explore novel antimicrobial strategies targeting stress pathways. This review synthesizes recent advances in the field, providing important reference value for optimizing clinical therapy, delaying the resistance crisis, guiding new drug development, and establishing integrated resistance surveillance (**Figure 4**).

**Figure 4:**
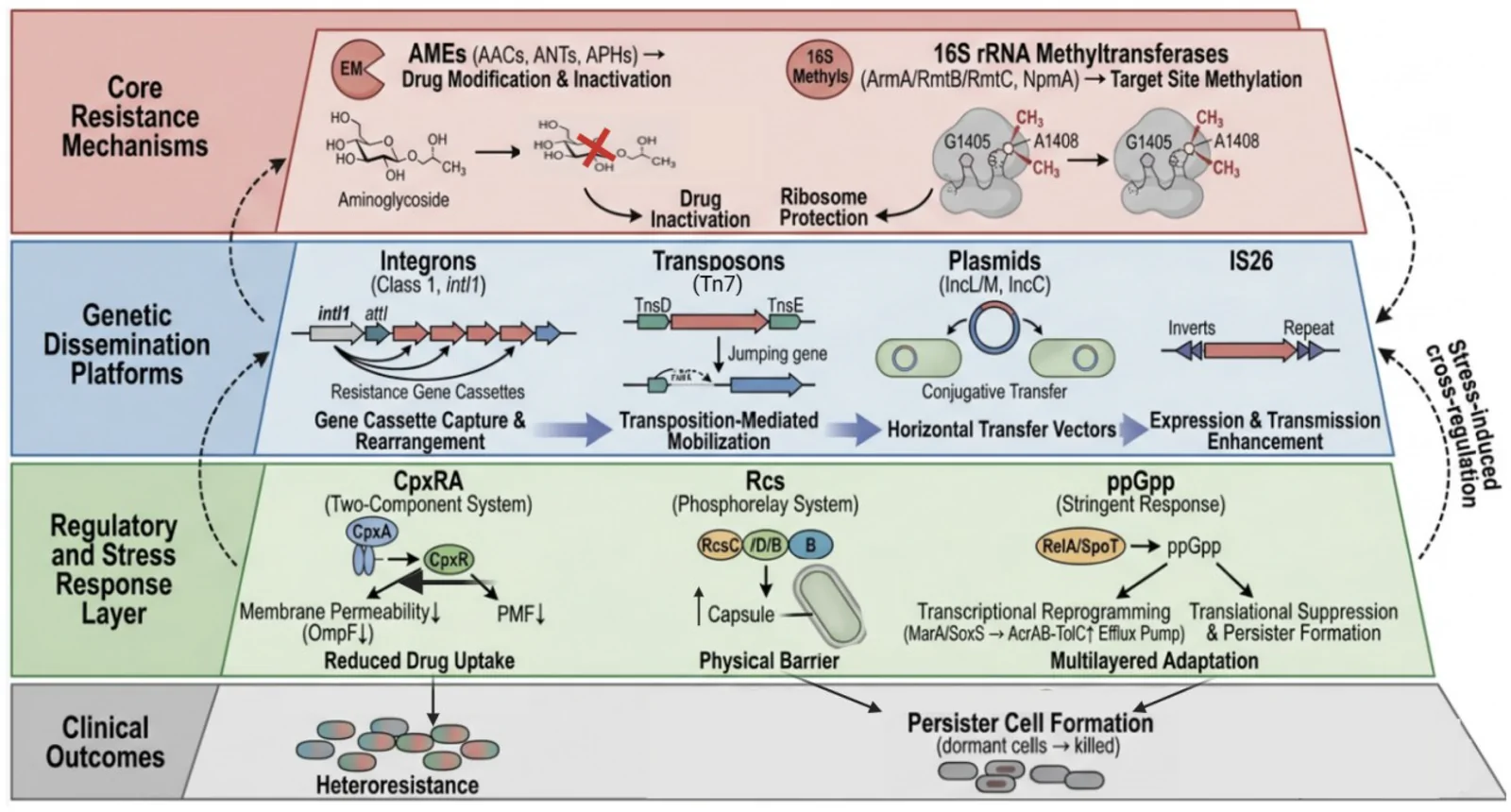
Integrated multilayer framework for aminoglycoside-resistance in clinical Enterobacteriaceae isolates.This schematic summarizes the hierarchical, interconnected resistance architecture in clinical isolates. At the core-resistance-mechanism layer, aminoglycoside-modifying enzymes (AMEs) catalyze drug inactivation, while 16S rRNA methyltransferases confer ribosome-target protection. At the genetic-dissemination platform layer, functionally distinct mobile genetic elements cooperate sequentially: integrons capture and rearrange resistance gene cassettes; transposons and IS26 mediate DNA mobilization; conjugative plasmids serve as major horizontal-transfer vectors for resistance determinants. At the regulatory-and-stress-response layer, intrinsic bacterial signalling cascades drive adaptive physiological defence. The CpxRA two-component system may decrease drug uptake by down-regulating OmpF porin and proton-motive force. The Rcs phosphorelay system may stimulate capsule biosynthesis to build physical barriers. The ppGpp-dependent stringent response can trigger transcriptional reprogramming, efflux-pump activation, translational suppression and persister-cell formation. Stress-induced cross-regulation establishes bidirectional crosstalk between mobile genetic elements and stress-response pathways. Collectively, these multilayer molecular events may contribute to heteroresistance and persister subpopulations, which may contribute to clinical treatment failure and infection relapse.
